# Generative AI use and advisory performance among agricultural extension agents in Benin

**DOI:** 10.3389/frai.2026.1763406

**Published:** 2026-05-26

**Authors:** Mori W. Gouroubera, Alcade C. Segnon, Morrisson Gouthon, Ismail Moumouni-Moussa, Robert B. Zougmoré

**Affiliations:** 1 International Center for Tropical Agriculture (CIAT), Dakar, Senegal; 2 Laboratory of Research on Innovation for Agricultural Development (LRIDA), Faculty of Agronomy, University of Parakou, Parakou, Benin; 3 Faculty of Agronomic Sciences, University of Abomey-Calavi, Cotonou, Benin

**Keywords:** agricultural extension, Artificial Intelligence in agriculture, behavioral intention, Benin, Generative AI, Technology Acceptance Model

## Abstract

Harnessing Generative Artificial Intelligence (GenAI) offers promising avenues to enhance agricultural advisory services. Yet, understanding extension agents' engagement with such technologies remains limited. Using the Technology Acceptance Model (TAM) as an analytical lens, this study investigates how agricultural extension agents in Benin interact with GenAI and its impact on their advisory performance. We surveyed 240 extension agents across six districts and applied Partial Least Squares Structural Equation Modeling to examine relationships among perceived usefulness, workload, time pressure, attitudes, behavioral intentions, GenAI use, and performance. Results reveal that GenAI use is positively associated with improved advisory effectiveness. Workload and pressure emerge as key motivators for GenAI use, while perceived usefulness strongly predicts both positive attitudes toward GenAI and perceived ease of use. However, contrary to TAM assumptions, attitude has a negative influence on behavioral intention, a paradoxical engagement pattern implying that while extension agents value GenAI, they hesitate to rely fully on it, reflecting concerns about professional judgment, accountability, and trust in AI outputs. Finally, attitude, pressure, and behavioral intention indirectly affect the performance of extension agents using GenAI. This study contributes to agricultural extension research and AI governance debates by revealing how professional intermediaries navigate tensions between technological promise and institutional responsibility, offering insights for capacity-building and policy frameworks that promote responsible AI integration.

## 1 Introduction

Agricultural extension remains a central mechanism for translating research into practice, supporting farmer productivity, resilience, and the scaling of sustainable farming practices across low- and middle-income contexts (Gebresilasse, 2023; Gouroubera et al., 2023a; Estefan et al., 2025; Nouatin et al., 2025; Obi et al., 2025). The ongoing digital transformation of agriculture, characterized by data-enabled advisory systems, ICT-enabled extension, and the emergence of AI-driven tools, offers opportunities to extend advisory reach and relevance in regions where extension capacity is limited and information demands are rising (Elbasi et al., 2023; Gouroubera et al., 2023b, 2025; Tzachor et al., 2023; Kpodo and Nejadhashemi, 2025; Shahriar et al., 2025). Generative Artificial Intelligence (GenAI), including conversational systems and large language model-driven assistants, has shown potential to generate context-specific responses, translate technical knowledge into local language forms, and assist in the production of advisory content that could complement traditional extension channels (Pandey and Mishra, 2024; Calone et al., 2025; Shahriar et al., 2025). These dynamics motivate closer scrutiny of how GenAI can be integrated into farm advisory services while acknowledging the practical constraints of public-extension systems in Africa and beyond (Calone et al., 2025; Kpodo and Nejadhashemi, 2025; Omotayo et al., 2025).

Recent literature shows that AI is rapidly transforming agriculture through applications in crop management, pest and disease detection, predictive analytics, and extension services (Elbasi et al., 2023; Ganeshkumar et al., 2023; Oliveira and Silva, 2023). Evidence highlights its strong potential to enhance productivity and sustainability (Sachithra and Subhashini, 2023; Shahriar et al., 2025). However, challenges persist, including limited data quality, poor accessibility, weak infrastructure, and ethical concerns (Oliveira and Silva, 2023; Abdelgadir Mohamed et al., 2024; Hagendorff, 2024; Omotayo et al., 2025). In the specific case of GenAI, concerns about hallucinations (the generation of inaccurate or misleading information), accountability for AI-assisted recommendations, and the governance of decision processes are especially salient for advisory contexts where risk management and professional judgment are central (Dara et al., 2022; Hagendorff, 2024). These barriers continue to constrain the equitable and effective adoption of AI innovations in agriculture, especially in developing countries.

Despite this growing application of AI-enabled agricultural innovations, there is a notable gap in understanding extension agents' behavioral engagement with GenAI in real-world advisory settings (Tzachor et al., 2023; Kpodo and Nejadhashemi, 2025). Most existing work focuses on farmer-facing ICT adoption or general ICT integration in extension, without explicitly examining extension intermediaries as those who translate innovations into practical guidance for farmers. Moreover, extension agents are not merely technology users; they are professionals operating within institutional hierarchies, performance regimes, and trust-based relationships with farmers (Birner et al., 2009). Their autonomy, accountability, and incentives are structured by governance arrangements, organizational cultures, and funding mechanisms (Birner et al., 2009). These institutional conditions make adoption dynamics among extension agents different from those observed among farmers, because agents must reconcile GenAI outputs with professional judgment, service standards, and the relational work of maintaining farmer trust (Birner et al., 2009; Alexander et al., 2024).

A “best fit” lens from pluralistic extension scholarship clarifies why context matters: performance depends on how governance, organizational capacities, advisory methods, and community engagement align with the policy environment and local farming systems (Birner et al., 2009; Klerkx, 2020). Furthermore, trust and professional judgment are central to advisory mediation. Organizational trust theory shows that willingness to depend on advice is shaped by perceptions of advisor ability, benevolence, and integrity (Mayer et al., 1995). In agricultural advisory systems, tailoring governance, methods, and engagement to local conditions improves performance and uptake, especially when advisors act as credible intermediaries (Birner et al., 2009; Klerkx, 2020). However, it remains unclear why and how GenAI introduces new challenges compared to earlier information and communication tools. Unlike static or rule-based advisory systems, GenAI can autonomously generate content, synthesize diverse knowledge sources, and provide recommendations that may influence professional judgment, accountability structures, and the localization of advice (Hagendorff, 2024; Calone et al., 2025). These capabilities raise questions about trust, control, and the governance of AI-assisted decisions, dimensions that existing studies have not sufficiently addressed (Tzachor et al., 2023; Omotayo et al., 2025). Given the expanding use of AI across multiple sectors, it is crucial to understand how extension agents perceive and interact with GenAI tools and the outcomes. Such understanding is essential because extension agents intervene as vital mediators between AI-enabled knowledge systems and farmers, and their attitudes toward GenAI can significantly influence advisory quality, equity of access, and the governance of AI-assisted decision-making.

This study addresses these gaps by examining the behavioral intentions and actual use of GenAI for farm advisory services among agricultural extension agents in Benin, West Africa. The research investigates how extension agents' beliefs translate into adoption behavior and subsequent performance outcomes in advisory delivery. By focusing on extension agents as the primary intermediaries, the study contributes to refining our understanding of AI adoption in agricultural knowledge systems.

## 2 Analytical framework

**Figure 1:**
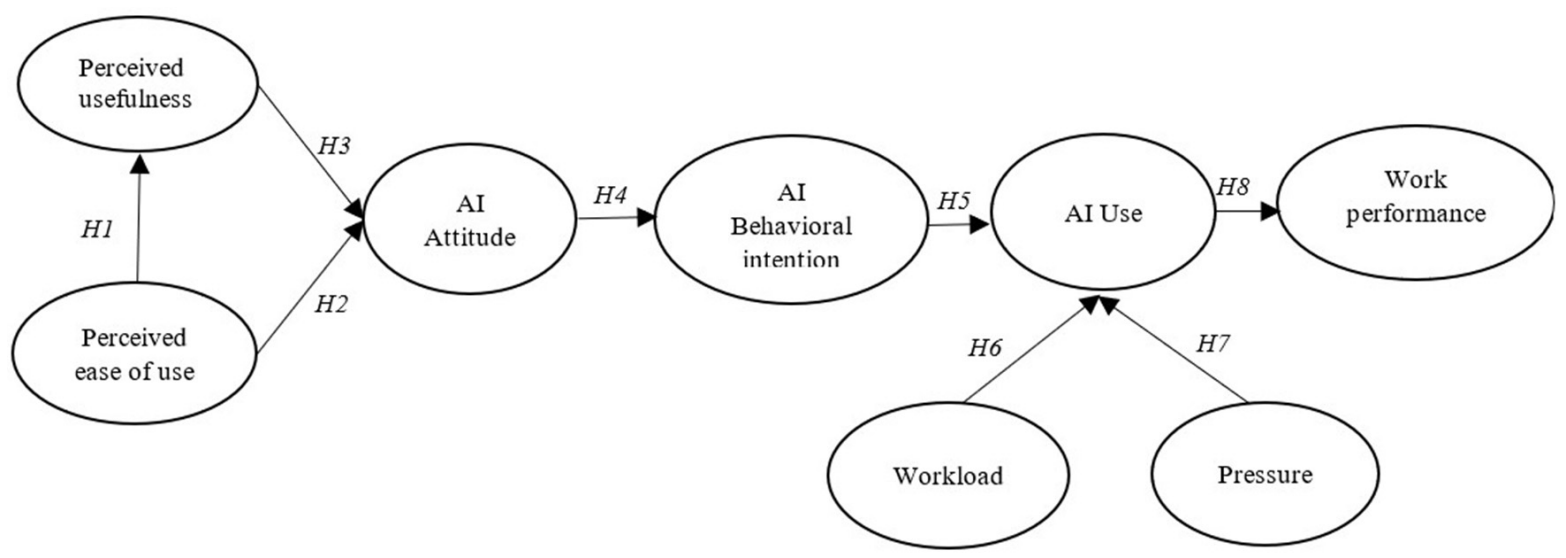
Analytical framework.

The Technology Acceptance Model (TAM) provides a foundational framework for understanding user adoption of technology, emphasizing perceived usefulness and perceived ease of use as key determinants of behavioral intention (Davis, 1989). TAM has been widely applied to explain how digital tools influence advisory practices (Venkatesh et al., 2003). While TAM offers a clear and familiar analytical structure, it has well-documented limitations in capturing the socio-technical and organizational conditions under which extension agents operate (Venkatesh et al., 2003; Klerkx et al., 2012). To address this, we extend TAM by integrating workload and pressure as contextual drivers and by situating GenAI adoption within necessity-driven and coping behaviors under systemic stress (Figure 1). This extension reflects the realities of extension work, where technology adoption may be motivated not only by perceived usefulness but also by efficiency imperatives and institutional performance regimes (Birner et al., 2009; Tarafdar et al., 2015; Califf et al., 2020).

TAM explains that an individual's decision to adopt a technology is determined primarily by its *perceived usefulness* (PU) and *perceived ease of use* (PEU) (Davis, 1989; Davis and Venkatesh, 1996). In the context of agricultural extension, *perceived usefulness* reflects the degree to which extension agents believe that using GenAI will enhance their advisory performance and service delivery efficiency. Prior studies have consistently shown that PU significantly influences users' attitudes and behavioral intentions toward digital technologies (Chen et al., 2025). Meanwhile, PEU represents the extent to which an individual expects using GenAI tools to be effortless. When a technology is easy to operate, users are more likely to develop positive attitudes toward it (Davis and Venkatesh, 1996). Furthermore, ease of use often influences perceptions of usefulness, as users who find AI simple to use are more likely to perceive it as beneficial (Davis and Venkatesh, 1996). Nevertheless, some studies have occasionally found that PU is positively associated with PEU (Toros et al., 2024). Consequently, we hypothesize that:

***H1:*** *Perceived ease of use has a positive effect on perceived usefulness*.

***H2:*** *Perceived ease of use has a positive effect on attitude toward using GenAI*.

***H3:*** *Perceived usefulness has a positive effect on attitude toward using GenAI*.

Attitude refers to an individual's overall positive or negative evaluation of using a particular technology. In line with TAM, attitude is a direct predictor of behavioral intention (BI), the motivational factor that determines an individual's likelihood of actual use (Davis, 1989). Recently, scholars have developed AI attitude scales (Grassini, 2023; Stein et al., 2024), offering deeper insights into individuals' perceptions and emotional orientations toward AI. In the context of agricultural extension services, when extension agents hold favorable attitudes toward AI, they are more likely to experiment with and adopt it in their farm advisory activities. Accordingly, we propose that:

***H4:*** *Attitude toward GenAI positively influences behavioral intention to use it*.

Behavioral intention has been widely validated as the strongest predictor of actual technology use (Venkatesh et al., 2003; Venkatesh, 2022; Stein et al., 2024). In the context of this study, intention reflects the willingness of extension agents to incorporate GenAI into their advisory activities. Stronger behavioral intentions are expected to lead to more frequent and sustained use of AI tools for delivering farm advice. Consequently, we suggest that:

***H5:*** *Behavioral intention to use GenAI positively influences its actual use*.

Beyond psychological determinants, contextual and organizational factors also influence technology use. Workload refers to the perceived volume and intensity of tasks that extension agents must complete, whereas pressure reflects the urgency or external demands to achieve work-related goals. Abbas et al. (2024) developed workload and pressure measurement scales related to ChatGPT usage among students, which were adapted for this study. Workload and pressure can have competing effects, not only on adoption but also on the quality of innovation outcomes. While heavy workloads may drive necessity-based adoption and efficiency-seeking behaviors, they can limit critical engagement with GenAI outputs, reducing opportunities for contextual adaptation and thorough validation. This tension suggests that systemic stress may accelerate uptake while constraining the depth and reliability of advisory recommendations. We argue that:

***H6:*** *Workload has a positive effect on GenAI use*.

***H7:*** *Pressure has a positive effect on GenAI use*.

In advisory contexts, GenAI use is expected to influence extension worker performance by improving information quality, decision speed, and accuracy of recommendations. Prior research has linked technology utilization to improved productivity and service effectiveness (Elbasi et al., 2023; Abbas et al., 2024). Thus, more frequent and effective use of GenAI should enhance extension agents' ability to deliver relevant and timely advice to farmers. Thus, we argue that:

***H8:*** *GenAI use positively affects extension agents' performance*.

In addition to the direct effects, several indirect pathways are proposed. Perceived usefulness, perceived ease of use, workload, and pressure are expected to influence GenAI use and performance indirectly through attitude or behavioral intention, consistent with TAM logic (Stein et al., 2024; Venkatesh, 2022). Moreover, attitude is hypothesized to mediate the relationships between perceptual factors and behavioral intention, while behavioral intention mediates the effects of attitude on actual use and performance.

***H9:*** *Attitude mediates the relationship between perceived usefulness and behavioral intention*.

***H10:*** *Behavioral intention mediates the relationship between attitude and GenAI use*.

***H11:*** *The use of GenAI mediates the relationship between behavioral intention and performance*.

***H12:*** *GenAI use mediates the relationship between workload and performance*.

***H13:*** *GenAI use mediates the relationship between pressure and performance*.

## 3 Methodology

### 3.1 Sample and study area

The study was conducted across six districts in northern Benin: Tchaourou, Banikoara, Malanville, Djougou, Natitingou, and Kèrou. The target population consisted of agricultural extension agents officially registered with the Agricultural Development Cluster Areas (ATDA—acronyms in French; there are seven ATDA in total, each focusing on a specific agricultural value chain) operating in the six districts. From the list of 320 officially registered extension agents in the three ATDA covering the six districts, a random sampling was employed to select 240 agents. The sample represents 75% of the registered extension agents in each district (Table 1).

**Table 1:** Distribution of the study sample.

Districts	Number of registered extension agents	Number of extension agents surveyed
Tchaourou	80	60
Banikoara	57	43
Malanville	53	40
Djougou	55	41
Natitingou	40	30
Kèrou	35	26
Total	320	240

Data were collected between March and May 2024 using a structured questionnaire administered through face-to-face interviews. The instrument was pre-tested with 15 extension agents in a non-sample district (N'Dali) to assess question clarity, reliability, and internal consistency. Necessary revisions were made before full implementation. Of the 240 respondents, 65% were male, and 35% were female. The average age of respondents was 33.74 years (SD = 6.40). Regarding institutional affiliation, 23% of the respondents were employed in the public sector, while 77% worked in private-sector organizations providing agricultural advisory services.

### 3.2 Measures

All items measuring the latent variables were measured using a 5-point Likert scale, ranging from 1 (strongly disagree) to 5 (strongly agree). The constructs included in the model were AI Perceived Usefulness, AI Perceived Ease of Use, AI Attitude, AI Behavioral Intention, extension agents' Workload, and extension agents' Pressure. The variable GenAI Use was measured as a single-item. Performance was measured as a single-item derived from four performance criteria: the number of farmers trained, advisory speed, precision and quality of recommendations, and relevance of the advisory content.

#### 3.2.1 AI perceived usefulness

This construct was measured using five items adapted from the Technology Acceptance Model (TAM) (Davis, 1989). Example items include “*Using AI tools such as ChatGPT helps me provide faster and more accurate advisory services to farmers”* and “*AI tools improve the quality and relevance of the agricultural information I deliver*. Higher scores indicate greater perceived usefulness of AI.

#### 3.2.2 AI ***perceived ease of use***

*The* PEU construct was measured with three items, also adapted from (Davis, 1989). Example statements include: “*Learning to use AI tools such as ChatGPT for extension work is easy for me”; “I find it simple to interact with AI tools when searching for agricultural information or preparing advisory messages”*.

#### 3.2.3 AI attitude

*Four* items were used to measure this construct, adapted from Grassini (2023) and Stein et al. (2024). Example items include “*I believe that using AI tools such as ChatGPT can improve the quality of my extension work”; “I believe that AI can make my work with farmers more efficient and effective”*.

#### 3.2.4 AI behavioral intention

Four items (Davis, 1989) was used to measure BI. Sample statements include “*I intend to regularly use AI-based tools in my work as an extension worker”; “I plan to increase my knowledge and skills related to AI applications for agricultural extension*.”

#### 3.2.5 Workload

It was measured using four items, adapted from previous studies related to AI usage workload scales (Abbas et al., 2024). Examples include “*My workload as an extension worker is too heavy; I feel overloaded by the number of tasks I must accomplish in my extension work.”*

#### 3.2.6 Pressure

It was measured with four items (Abbas et al., 2024). Statements included “*I often do not have enough time to prepare for my farmer training sessions or field visits”; “I do not have sufficient time to complete my extension tasks with the level of quality I would like*.”

#### 3.2.7 AI use

This was measured using one item (Matthews et al., 2022). Respondents indicated how often they used AI for specific tasks such as designing training content, answering farmers' questions, or searching for agricultural information to advise farmers.

#### 3.2.8 Performance

Extension performance was objectively measured to minimize self-report and social desirability bias. Extension agents did not directly report their performance. We asked their supervisor to report their last 6-monthly rate of performance, measured considering the following criteria: the number of farmers trained, speed, precision, and relevance of their advisory, as officially recorded in the ATDA performance assessments guide^1^. Supervisors were defined as the immediate line managers within the organization's structure responsible for monitoring and evaluating the activities of extension agents. Each supervisor evaluated one to five extensions, consistent with the standard reporting procedures of the ATDA system. To enhance consistency across evaluators, all supervisors rely on a common institutional performance assessment guide, which specifies standardized criteria and rating scales for evaluating extension activities. The average score of the four criteria was used to form the performance score, which ranged from 1 (very low) to 7 (excellent). Although the measure relies on supervisory judgment, the use of standardized institutional guidelines helps ensure a degree of comparability across evaluations. As this variable was collected as a single objective rating, no reliability or validity tests were required. However, we acknowledge that some degree of subjectivity may remain due to evaluator-specific judgment.

### 3.3 Measurement model

We conducted Partial Least Squares Structural Equation Modeling (PLS-SEM) in SmartPLS 4.1.1.5 software to assess the measurement model's reliability and validity. PLS-SEM is suitable for predictive and exploratory modeling involving complex relationships among latent constructs (Hair et al., 2019). The assessment proceeded in two stages: the measurement model and the structural model. In the first stage, construct reliability and validity were examined to ensure that the latent variables were measured reliably. Construct reliability was evaluated using Cronbach's Alpha and Composite Reliability (ρ_A_ and ρ_C_), with values above 0.70 considered satisfactory. Convergent validity was assessed through the Average Variance Extracted (AVE), which should exceed 0.50 for each construct (Hair et al., 2019). As shown in Table 2, the standardized factor loadings of the retained items were above 0.70, except for one item in AI perceived ease of use (0.697). Both Cronbach's alpha and Composite Reliability values exceeded the 0.70 threshold, and AVE values were greater than 0.50, confirming adequate internal consistency and convergent validity (Sarstedt et al., 2019). During the analysis, two items were dropped and excluded from the analysis: one attitude item with a factor loading of 0.671 and one behavioral intention item with a factor loading of 0.669. Although these loadings were close to the required threshold (0.70), their removal improved the reliability of the constructs.

**Table 2:** Reliability and validity of the construct.

Constructs with their items	Factor loadings	Cronbach Alpha	ρ_A_	ρ_C_	AVE
AI attitude		0.752	0.785	0.857	0.668
I believe that using AI tools such as ChatGPT will improve the quality of my extension work	0.775	
I believe that AI will make my work with farmers more efficient and effective	0.671 (dropped)	
I think I will use AI tools in my extension activities in the future	0.809	
I believe that the increasing use of AI in agricultural advisory services may negatively affect human extension agents ***(reverse-coded item)***	0.865	
AI Behavioral Intention		0.879	0.913	0.925	0,803
I intend to regularly use AI-based tools in my work as an extension worker	0.922	
I plan to increase my knowledge and skills related to AI applications for agricultural extension.	0.891	
I will recommend the use of AI technologies to farmers and colleagues in the near future	0.669 (dropped)	
Whenever I have access to AI tools, I will use them to improve the quality of my extension services	0.875	
AI Perceived Ease of Use		0.743	0.763	0.856	0.666
Learning to use AI tools such as ChatGPT for extension work is easy for me	0.856	
I find it simple to interact with AI tools when searching for agricultural information or preparing advisory messages	0.884	
It would be easy for me to become skilled at using AI tools in my daily extension activities	0.697	
AI Perceived Usefulness		0.801	0.803	0.863	0.558
Using AI tools such as ChatGPT helps me provide faster and more accurate advisory services to farmers	0.77	
AI tools improve the quality and relevance of the agricultural information I deliver	0.778	
Using AI reduces the time and effort needed to prepare extension messages and training materials	0.701	
AI tools enhance my ability to find solutions to farmers' problems	0.748	
Using AI makes me more effective in performing my duties as an extension worker	0.734	
Pressure		0.902	0.967	0.93	0.768
I often do not have enough time to prepare for my farmer training sessions or field visits	0.863	
I do not have sufficient time to complete my extension tasks with the level of quality I would like	0.866	
I find it difficult to meet the deadlines for submitting my extension reports or activity plans	0.895	
I am often in a hurry to meet multiple work deadlines related to my extension duties	0.882	
Workload		0.914	0.944	0.938	0.792
My workload as an extension worker is too heavy	0.933	
I feel overloaded with the number of tasks I must accomplish in my extension work	0.934	
I often feel overburdened by the number of responsibilities in my extension work	0.913	
The management assigns too many activities and targets for me to achieve within a short time	0.769	

Discriminant validity was established using the Fornell-Larcker criterion and the Heterotrait-Monotrait (HTMT) ratio. Discriminant validity is achieved when the square root of the AVE for each construct (diagonal elements) is greater than its correlations with other constructs (Fornell and Larcker, 1981). This condition was acceptable for all constructs (Table 3). Additionally, the HTMT ratios were examined, with most values falling below the recommended threshold of 0.85 (Table 4). Although the correlation between pressure and behavioral intention is relatively high (0.854), the square root of the AVE for both constructs exceeds this value, indicating that discriminant validity is established. Overall, the measurement model demonstrated satisfactory reliability, convergent validity, and discriminant validity. These results confirm that the constructs met the required criteria for inclusion in the final PLS-SEM structural model examining extension agents' adoption and behavioral use of GenAI tools.

**Table 3:** Discriminant validity (Fornell–Larcker criterion).

Constructs	AI attitude	AI behavioral intention	AI perceived ease of use	AI perceived usefulness	Performance	Pressure	AI use	Workload
AI attitude	**0.817**							
AI behavioral intention	−0.429	**0.896**						
AI perceived ease of use	0.478	−0.562	**0.816**					
AI perceived usefulness	0.705	−0.597	0.731	**0.747**				
Performance	0.271	−0.305	0.514	0.475	**1**			
Pressure	0.541	−0.757	0.65	0.667	0.378	**0.877**		
AI use	0.234	−0.028	0.367	0.303	0.411	0.169	**1**	
Workload	0.607	−0.659	0.624	0.69	0.424	0.737	0.165	**0.89**

Diagonal values in bold represent the square root of the AVE.

**Table 4:** Discriminant validity [Heterotrait–Monotrait (HTMT) ratio].

Constructs	AI attitude	AI behavioral intention	AI perceived ease of use	AI perceived usefulness	Performance	Pressure	AI use
AI attitude							
AI behavioral intention	0.514						
AI perceived ease of use	0.609	0.691					
AI perceived usefulness	0.845	0.699	0.841				
Performance	0.302	0.318	0.594	0.533			
Pressure	0.645	0.854	0.788	0.776	0.396		
AI use	0.258	0.037	0.42	0.343	0.411	0.171	
Workload	0.729	0.746	0.753	0.815	0.425	0.84	0.163

## 4 Results

After validating the measurement model, we tested the study hypotheses to examine both direct and indirect relationships among constructs. The structural model was estimated using the bootstrapping procedure with 5,000 resamples to ensure robust significance testing of path coefficients (Hair et al., 2019). Although our hypothesis H1(PEU → PU) was formulated following the original TAM logic (Davis and Venkatesh, 1996), the estimated model revealed the reverse path (PU → PEU), which we treat as a post-hoc exploratory finding. To further assess the structural model, we examined the variance inflation factor (VIF) values of all predictor constructs to check for collinearity. Table 5 shows that all VIF values are below the conservative threshold of 3.3 indicating no concern about collinearity (Diamantopoulos and Siguaw, 2006). The explanatory power of the model was assessed using R^2^ values according to absolute standards (Hair et al., 2017). PEU (*R*^2^ = 0.535) and attitude (*R*^2^ = 0.500) indicate moderate explanatory power. In contrast, behavioral intention (*R*^2^ = 0.184) and performance (*R*^2^ = 0.169) show weak explanatory power, while AI use (*R*^2^ = 0.061) is only marginally explained. Although the model's in-sample explanatory power is relatively low in absolute terms, it is considered acceptable given the model's complexity and the exploratory nature of the study. However, it should be interpreted under this limitation. The predictive performance of the model was assessed using PLSpredict with 10-fold cross-validation and 10 repetitions (Shmueli et al., 2019). The results indicate heterogeneous predictive accuracy across constructs. PEU (${\text{Q}}_{\text{predic}\text{t =}}^{2}$0.646) and attitude (${\text{Q}}_{\text{predic}\text{t =}}^{2}$0.484) show strong predictive relevance, while behavioral intention (${\text{Q}}_{\text{predic}\text{t =}}^{2}$0.258) and AI use (${\text{Q}}_{\text{predic}\text{t =}}^{2}$0.130) shows moderate predictive performance. In contrast, performance (${\text{Q}}_{\text{predic}\text{t =}}^{2}$0.080) demonstrates weak but positive predictive relevance. Overall, the model indicates strong predictive capability for perceptual and attitudinal constructs and moderate predictive power for behavioral outcomes. Therefore, we conclude that the model has satisfactory out-of-sample predictive power, particularly for perceptual constructs. Furthermore, we examined effect sizes (f^2^) to assess the relative impact of exogenous constructs on endogenous variables (Hair et al., 2019). Results indicated that PU had a large effect on PEU (f^2^ = 1.859) and attitude (f^2^ = 0.514). Attitude had a medium effect on behavioral intention (f^2^ = 0.226). GenAI use had also a medium effect on performance (f^2^ = 0.203). In contrast, PEU (f^2^ = 0.125), behavioral intention (f^2^ = 0.131), workload (f^2^ = 0.090), and pressure (f^2^ = 0.024) showed small effects. The global model fit was assessed using the Standardized Root Mean Square Residual (SRMR). The SRMR value was 0.058 which is below the recommended threshold of 0.08 (Hair et al., 2019), indicating that the model provides an adequate approximation of the observed data.

**Table 5:** Assessment of the structural model.

Path	VIF	*R^2^*	Q^2^ predict	f^2^	SRMR
AI use → performance	1	0.169	0.08	0.203	0.058
AT → BI	1	0.184	0.258	0.226	
BI → AI use	2.476	0.061	0.13	0.131	
Workload → AI use	2.307			0.09	
Pressure → AI use	3.062			0.024	
PEU → AT	2.859	0.500	0.484	0.131	
PU → AT	2.859			0.514	
PU → PEU	1	0.535	0.646	1.859	

**Figure 2:**
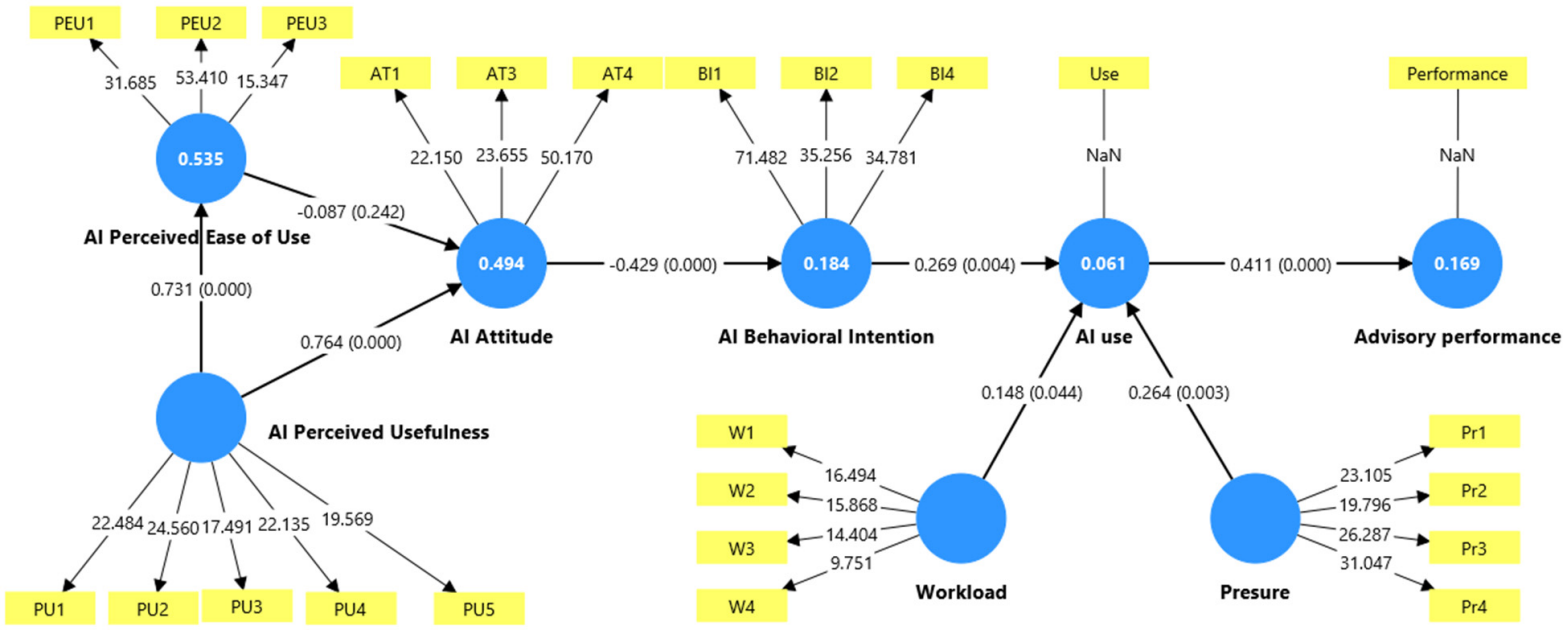
Structural model.

Figure 2 illustrates the final structural model along with the path coefficients and *p*-values based on two tailed tests. The results indicate that Perceived Usefulness is positively associated with Attitude toward GenAI (β = 0.764, *t* = 12.226, *p* < 0.001). Similarly, Perceived Usefulness is positively associated with Perceived Ease of Use (β = 0.734, *t* = 22.617, *p* < 0.001).

Contrary to expectations, Perceived Ease of Use did not significantly influence Attitude (β = −0.085, *t* = 1.171, *p* = 0.242). Moreover, the link between Attitude and Behavioral Intention was negative and significant (β = −0.429, *t* = 8.172, *p* < 0.001). This unexpected finding may indicate cautious orientation among extension agents. Extension agents who express strong attitudes toward GenAI might simultaneously experience professional identity threats, heightened accountability concerns, or fears of misaligned advice that could undermine trust with farmers. These factors could lead to cautious behavioral intentions despite positive attitudes. As hypothesized, Behavioral Intention is positively associated with GenAI Use (β = 0.269, *t* = 2.915, *p* = 0.004), implying that stronger intentions translate into more frequent use of GenAI tools in extension activities. Work Pressure (β = 0.264, *t* = 3.026, *p* = 0.003) and Workload (β = 0.148, *t* = 1.920, *p* = 0.044) both positively affected GenAI Use. Extension agents under greater time or task constraints are more likely to use GenAI to manage their workload and meet performance expectations efficiently. Moreover, GenAI Use is positively associated with Extension Performance (β = 0.411, *t* = 8.229, *p* < 0.001), implying that greater utilization of GenAI is more likely to contribute to improved work outcomes, such as better farmer advisory results or faster service delivery.

Table 6 presents standardized path coefficients (β), standard deviations, t-statistics, and *p*-values of the total indirect effects. Several significant mediation pathways to performance were identified. First, the results show that GenAI use is a key mediating mechanism linking extension agents' work demands to performance outcomes. Work pressure (β = 0.108, *t* = 2.717, *p* = 0.005) has a significant and positive indirect effect on performance using GenAI. When extension agents face higher pressure, they are more likely to use GenAI tools for efficiency, which in turn improves their performance. Second, behavioral intention emerges as a driver of performance via GenAI use. Behavioral intention has a strong positive indirect effect on performance through GenAI Use (β = 0.111, *t* = 2.68, *p* = 0.008). Third, the path AT → BI → Use → Performance (β = −0.047, *t* = 2.441, *p* = 0.015) reinforces that unfavorable attitudes may constrain performance indirectly.

**Table 6:** Total indirect effects.

Path	Coef	SD	*t*	*P*-values	Hypotheses
PU → PEU → AT	−0.063	0.055	1.114	0.253	Rejected
PEU → AT → BI	0.037	0.032	1.104	0.117	Rejected
Pressure → AI use → performance	0.108	0.042	2.717	0.007	Accepted
PU → AT → BI	−0.328	0.050	6.581	0.000	Accepted
AT → BI → AI use	−0.15	0.043	2.692	0.007	Accepted
Workload → AI use → performance	0.064	0.033	1.882	0.060	Rejected
PU → PEU → AT → BI → AI use	0.007	0.008	0.988	0.323	Rejected
PEU → AT → BI → AI use	0.01	0.01	1.011	0.312	Rejected
PU → AT → BI → AI use	−0.088	0.034	2.576	0.010	Accepted
AT → BI → AI use → performance	−0.045	0.019	2.441	0.015	Accepted
PU → PEU → AT → BI	0.027	0.023	1.141	0.254	Rejected
PEU → AT → BI → AI use → performance	0.004	0.004	0.902	0.322	Rejected
BI → AI use → performance	0.111	0.041	2.681	0.008	Accepted
PU → AT → BI → AI use → performance	−0.038	0.016	2.345	0.019	Accepted
PU → PEU → AT → BI → AI use → performance	0.003	0.003	0.970	0.332	Rejected

## 5 Discussion

The emergence of GenAI is transforming various sectors, including agricultural advisory systems. While there has been considerable discussion about the effectiveness of GenAI in agriculture, recent literature has not addressed how extension agents can leverage GenAI to improve performance in achieving food security (Calone et al., 2025; Shahriar et al., 2025). Our study reveals the mechanisms through which GenAI can influence the effectiveness of agricultural advisory systems in low- and middle-income contexts.

### 5.1 GenAI as a decision-support system in agricultural extension, enabling adaptive learning

This study finds that the use of GenAI is positively associated with extension agents' performance, including the speed, precision, and relevance of their advisory services. These findings suggest that GenAI may function as a decision-support system that can complement, rather than replace, human expertise like AI-driven diagnostic tools in precision agriculture (Elbasi et al., 2023; Shahriar et al., 2025). GenAI appears to support extension agents to synthesize vast bodies of agricultural knowledge, simulate farmer inquiries, and deliver localized, context-specific advice efficiently. The positive association between GenAI use and performance is consistent with the argument that AI enhances real-time decision-making by generating context-aware recommendations (Duan et al., 2019; Al-Surmi et al., 2022). In resource-constrained extension systems such as in Benin, GenAI may act as a potential knowledge support tool, helping extension agents manage cognitive overload while improving the consistency and quality of their recommendations. Extension agents often process complex information, manage multiple tasks, and make time-sensitive decisions in advisory service delivery.

The findings also call for a reframing of technology acceptance within extension systems. Results indicate that perceived usefulness is the dominant driver of favorable attitudes toward GenAI, confirming long-standing assertions from the TAM (Davis, 1989; Davis and Venkatesh, 1996; Venkatesh et al., 2003). Extension agents who perceive AI as a tool that enhances their efficiency and service quality tend to report more favorable attitudes toward its use. However, the absence of a significant relationship between perceived ease of use and attitude suggests that for technically trained extension agents, functionality and utility may be more salient than usability. This likely reflects the nature of their professional role and task complexity, where delivering accurate and timely advice to farmers takes precedence over interface simplicity. In such contexts, agents may prioritize tools that enhance performance and decision quality rather than those that are merely easy to operate. This idea is reinforced by the positive association observed between perceived usefulness and perceived ease of use, which departs from the original TAM specification. This deviation should be interpreted cautiously, as it may reflect context-specific perceptions. It suggests that extension agents may first evaluate whether GenAI is useful for their professional tasks before perceiving it as easy to use. This contrasts with findings among farmers and students, where ease of use is often a stronger predictor of adoption (Dwivedi et al., 2019; Abbas et al., 2024).

Unexpectedly, the negative relationship found between attitude and behavioral intention should be interpreted with caution. While it may indicate an underlying ambivalence among extension agents, this finding could also reflect measurement limitations. As extension agents, they are not simply technology adopters but operate within institutional hierarchies, performance evaluation systems, and trust-based relationships with farmers. These dynamics create strong accountability pressures, where inaccurate or misaligned advice can damage professional credibility and erode farmer trust. Therefore, it is plausible that even agents with favorable attitudes may report restrained behavioral intentions, although further research is needed to confirm this mechanism. This tension underscores that adoption decisions are shaped not only by perceived technological benefits but also by broader sociological factors such as professional identity, responsibility norms, and the imperative to maintain trust in advisory relationships. Also, while they acknowledge GenAI's potential benefits, some may recognize its limitations in providing localized and context-specific recommendations. Research indicates that while ChatGPT can handle general queries, it struggles with site-specific agronomic advice, such as planting time, seed rate, and fertilizer application, which remain critical for smallholder farmers (Cong-Lem et al., 2024; Ibrahim et al., 2024). Similar concerns have been raised in other contexts, where large language models exhibit bias and lack domain-specific reliability (Prestegaard-Wilson and Vitale, 2024). Such considerations may contribute to cautious or selective use rather than full adoption.

Interestingly, workload and job pressure were found to positively influence GenAI use, indicating that technology adoption may also be necessity-driven rather than purely motivational. When under pressure to meet reporting deadlines or serve large farmer populations, extension agents appear more likely to report using GenAI tools, possibly as a coping mechanism. This pattern aligns with previous research showing that digital stressors can paradoxically stimulate adaptive technology use when users perceive innovation as a coping resource (Tarafdar et al., 2015; Sinha et al., 2025). Thus, scaling GenAI in advisory systems may occur not only through top-down institutional mandates but also through bottom-up, stress-induced adaptation, especially when the technology demonstrably reduces task complexity and administrative burden.

### 5.2 Limitations and future research avenues

The use of GenAI among extension agents may be associated with agricultural technology scaling and broader food system transformation (Estefan et al., 2025; Nouatin et al., 2025). As extension agents integrate GenAI into their routines, there may be potential for improved advisory efficiency, which could support the diffusion of sustainable agricultural practices. This integration could contribute to a digitally mediated innovation system that bridges gaps in knowledge equity and service coverage. Yet, this potential will depend on institutional investments addressing digital literacy, data ethics, and algorithmic transparency (Gebresilasse, 2023; Obi et al., 2025). Beyond technical considerations, questions of accountability remain critical. Uncertainty around responsibility for AI-informed recommendations may influence agents' willingness to rely on GenAI, particularly in contexts where trust and professional credibility are central. Governance frameworks should therefore clarify accountability structures and ensure transparency to support responsible use.

The study focuses on extension agents in northern Benin, and findings may not be fully generalizable to other contexts. Comparative studies across multiple countries or extension systems would therefore enrich understanding of contextual influences on AI adoption among extension agents. Moreover, this research concentrated on individual-level factors, while organizational-level enablers, such as managerial support, data infrastructure, and ethical guidelines, were not included but are likely critical in shaping sustainable GenAI use. Additionally, the cross-sectional design of the study limits causal inference, and the relatively low explained variance for AI use suggests that other factors may have been missed. Furthermore, both GenAI use and performance were measured with single items, which limits the ability to model measurement error. Future research should employ multi-item constructs and longitudinal designs to improve the understanding of how GenAI transforms agricultural advisory systems, the evolving role of human expertise, and the design of responsible AI ecosystems for rural development.

## 6 Conclusion

This study provides empirical evidence on the behavioral mechanisms driving extension agents' use of GenAI for agro-advisories. By integrating technology acceptance factors with work-related demands, the results suggest that perceived usefulness is more strongly associated with favorable attitudes than perceived ease of use, indicating the importance of task relevance in professional contexts. At the same time, the study reveals a nuanced adoption process: although favorable attitudes are observed, they do not necessarily translate into stronger behavioral intentions, which may reflect accountability concerns, professional identity, or measurement limitations. This ambivalence illustrates that technology adoption among extension agents is not purely a technical decision but is embedded within institutional, ethical, and relational dynamics. Importantly, workload and work pressure are positively associated with GenAI use, suggesting that GenAI may serve as a coping or support tool under conditions of high demand, ultimately contributing to improved performance outcomes. Behavioral intention and actual GenAI use play a key mediating role in translating both work demands and perceptions into performance gains. This study advances understanding of GenAI adoption in agricultural extension by moving beyond simple acceptance models to reveal the complex interplay that may exist between perceived benefits, professional norms, and work pressures. The findings suggest that scaling GenAI in extension systems requires not only investment in digital tools but also supportive institutional frameworks that address trust, accountability, and ethical use. When appropriately governed, GenAI has the potential to support advisory systems, although further longitudinal and experimental research is needed to assess its actual impact on performance and agricultural outcomes.

## Data Availability

The original contributions presented in the study are included in the article/supplementary material, further inquiries can be directed to the corresponding author.

## Footnotes

1 The Ministry of Agriculture, Livestock, and Fisheries (MAEP) of Benin developed a national guide titled “Guide de contrôle du dispositif du conseil agricole par les DDAEP en République du Bénin” to monitor the delivery of agricultural extension services and serve as a tool for performance assessment.
